# The Structure of Ca^2+^ Sensor Case16 Reveals the Mechanism of Reaction to Low Ca^2+^ Concentrations

**DOI:** 10.3390/s100908143

**Published:** 2010-08-30

**Authors:** Lukas Leder, Wilhelm Stark, Felix Freuler, May Marsh, Marco Meyerhofer, Thomas Stettler, Lorenz M. Mayr, Olga V. Britanova, Lydia A. Strukova, Dmitriy M. Chudakov, Ekaterina A. Souslova

**Affiliations:** 1 Novartis Pharma AG, NIBR/CPC/LFP, Lichtstrasse 35, CH-4002 Basel, Switzerland; E-Mails: lukas.leder@novartis.com (L.L.); wilhelm.stark@intergga.ch (W.S.); felix.freuler@novartis.com (F.F.); may.marsh@novartis.com (M.M.); marco.meyerhofer@novartis.com (M.M.); thomas.stettler@novartis.com (T.S.); lorenz.mayr@novartis.com (L.M.M.); 2 Fachhochschule Nordwestschweiz, Institute for Chemistry and Bioanalytics. Gruendenstrasse 40, CH-4132 Muttenz, Switzerland; 3 Novartis Pharma AG, NIBR/CPC/EPP, Lichtstrasse 35, CH-4002 Basel, Switzerland; 4 Shemiakin-Ovchinnikov Institute of Bioorganic Chemistry, RAS, Miklukho-Maklaya str. 16/10, 117997, Moscow, Russia; E-Mails: Britanova@em.mpg.de (O.V.B.) lidda@list.ru (L.A.S.); chudakovdm@mail.ru (D.M.C.)

**Keywords:** circularly permuted green fluorescent protein, genetically encoded, fluorescent calcium indicator protein, crystal structure, calcium sensor

## Abstract

Here we report the first crystal structure of a high-contrast genetically encoded circularly permuted green fluorescent protein (cpGFP)-based Ca^2+^ sensor, Case16, in the presence of a low Ca^2+^ concentration. The structure reveals the positioning of the chromophore within Case16 at the first stage of the Ca^2+^-dependent response when only two out of four Ca^2+^-binding pockets of calmodulin (CaM) are occupied with Ca^2+^ ions. In such a “half Ca^2+^-bound state”, Case16 is characterized by an incomplete interaction between its CaM-/M13-domains. We also report the crystal structure of the related Ca^2+^ sensor Case12 at saturating Ca^2+^ concentration. Based on this structure, we postulate that cpGFP-based Ca^2+^ sensors can form non-functional homodimers where the CaM-domain of one sensor molecule binds symmetrically to the M13-peptide of the partner sensor molecule. Case12 and Case16 behavior upon addition of high concentrations of free CaM or M13-peptide reveals that the latter effectively blocks the fluorescent response of the sensor. We speculate that the demonstrated intermolecular interaction with endogenous substrates and homodimerization can impede proper functioning of this type of Ca^2+^ sensors in living cells.

## 1. Introduction

The development of effective fluorescent Ca^2+^ indicator proteins (FCIPs) is a challenge for a number of laboratories working with fluorescent proteins (FPs). Attempts to generate a sensor that combines high fluorescence brightness, fast and high-contrast response, low pH-dependency, does not interact with intracellular proteins, reliably targets specific cellular compartments, has high expression levels, and has the ability to reliably monitor Ca^2+^ changes in various systems (including neurons *in vivo* and *ex vivo*) has resulted in sequential improvements of FCIPs in recent years. However, a sensor combining all the properties mentioned above still has not been developed.

The most popular FCIP design uses distance and dipole orientation changes between the donor and acceptor FP chromophores mediated by Ca^2+^-dependent conformational changes of fused Ca^2+^-sensitive domains (such as calmodulin (CaM) [1–8] or troponin C (TnC) [9–11]), leading to more or less pronounced changes in the Förster Resonance Energy Transfer (FRET) efficiency. An alternative approach is to insert the single circularly permuted green fluorescent protein (cpGFP) between CaM and M13-peptide (fragment of myosin light-chain kinase). The circular permutation of GFP-like proteins allows for the fusion of sensing domains in the close proximity to the chromophore. Thus Ca^2+^-dependent conformational changes can influence the chromophore environment and the fluorescent properties of the sensor directly. A number of FCIPs of the latter type, employing cpGFPs at positions 145–148 (such as Pericams [5] and GCaMPs [2,4,6,8]), have been previously reported. The cpGFP-based FCIPs with the highest contrast described to date are 12- and 16-fold contrast Ca^2+^ sensors Case12 and Case16 [7]. They are characterized by high brightness, fast maturation at 37 °C and pronounced fluorescence changes in response to hundreds of nanomoles of Ca^2+^. Despite the fact that cpGFP-based FCIPs have a mechanism essentially identical to those of so-called “photo-activatable” FPs (*i.e.*, the deprotonation of the neutral GFP chromophore), the contrast of the best FCIPs developed to date—GCaMP2 [6], GCaMP3 [8], Case12 and Case16 [7]—is only 5–16-fold, while photoactivatable FPs can reach 100–400 fold contrast levels [12,13]. Thus it is reasonable to assume that the fluorescent response of cpGFP-based FCIPs can still be improved.

Progress in the generation of improved FCIP variants has been limited by the absence of structural data describing cpGFP alone and fused with Ca^2+^-sensitive domains. In most of cpGFP-based FCIPs the permutation point (*i.e.*, the breakpoint of the polypeptide chain) is located between amino acids 145 and 148 in the native sequence. However, until recently the real spatial positioning of the chromophore environment as well as relative positioning of the linkers, cpGFP fluorescent “core” and fused Ca^2+^-sensitive domains remained unclear. Thus the structural information about cpGFP-based Ca^2+^ sensors is absolutely crucial for their further improvement as well as for development of cpGFP-based sensors for analytes other than Ca^2+^.

Recently two research groups independently published the crystal structure of the Ca^2+^ sensor GCaMP2 in its Ca^2+^-saturated form [14,15]. These data revealed the relative arrangement of domains and a number of key features of the chromophore environment and allowed to enhance brightness and dynamic range of GCaMP2 using site-specific mutagenesis by decreasing solvent access to the chromophore. However, understanding the molecular details of the mechanism of the sensor response at low Ca^2+^ concentrations would be useful in the rational design of enhanced sensor variants.

Here we report the crystal structure of the high-contrast GCaMP-like (see Supplementary Figure 1 for protein sequence alignment) Ca^2+^ sensor Case16 [7] in the presence of low Ca^2+^ concentration. At this intermediate stage of Ca^2+^-dependent response Case16 is characterized by incomplete interaction of CaM with its target M13-peptide and its chromophore environment differs significantly from that of GCaMP2 in its Ca^2+^-saturated form reported earlier [14,15]. We also resolved the structure of the related Ca^2+^ sensor variant Case12 [7] at high Ca^2+^ concentration. This structure confirms that at high concentrations GCaMP-like Ca^2+^ sensors form non-functional homodimers that is consistent with the previously reported data for GCaMP2 [14,15]. Taken together, our data contribute to the structural understanding of cpGFP-based Ca^2+^ sensors and could enable the development of improved genetically encoded sensor variants for Ca^2+^ and other analytes.

## 2. Experimental Section

### 2.1. Cloning and Protein Purification

The DNA coding sequences of Case12 and Case16 sensors were amplified by PCR from the corresponding pQE30-based expression vectors (Qiagen) as described in [7]. For amplification of cDNA inserts a “sticky-end” PCR [17] was performed using a pair of 5′-end primers complementary to M13-peptide (5′-CTCACGTCGTAAGTGGAA-3′; 5′-GATCCTCACGTCGTAAGTGGAA-3′) and a pair of 3′-end primers complementary to CaM (5′-GGCCGCATTATTTTGCAGTCATCATCT GTACG-3′; 5′-GCATTATTTTGCAGTCATCATCTGTACG-3′) containing *Bam HI* and *Not I* restriction sites, respectively. To obtain the final expression vectors the coding sequence of the corresponding sensor variant was cloned using *BamHI/NotI* restrictions sites into reengineered expression vector pET41a(+) (Merck Biosciences) where the GST-tag has been deleted and the thrombin site was replaced by N-terminal fusion partner containing a His_6_-tag followed by a S-tag and a PreScission protease cleavage site [18].

Each of the plasmids coding either Case12 or Case16 sensor variant was transformed into BL21(DE3) Tuner *E. coli* cells. The bacteria were grown in TB mod cultivation medium containing 0.1 M MOPS buffer and 30 mg/L kanamycin to an OD_600_ = 0.8. For the induction of protein expression 0.1 mM IPTG was added and cells were incubated overnight at 20 °C. The cell pellets were harvested by centrifugation, resuspended in IMAC buffer A (50 mM Na-phosphate, 300 mM NaCl, 20 mM imidazole, pH 8.0) and lysed with a high pressure homogenizer (Avestin). The filtered lysate containing soluble proteins was loaded on a 5 mL His-Trap IMAC column mounted on an AEKTA Explorer 100 chromatography system (GE Healthcare). After extensive washing of the column with IMAC buffer A, 500 U of PreScission protease (GE Healthcare) were loaded onto the His-Trap column for direct cleavage of the N-terminal His6 S-tag fragment from the fusion protein. After further incubation at 4 °C during 10 h the cleaved Case12 and Case16 sensor proteins were eluted from the column with IMAC buffer A. Pooled fractions were subjected to size-exclusion chromatography that was performed on a Superdex75 XK16/60 column (GE Healthcare) using TBS running buffer (50 mM Tris-HCl, 150 mM NaCl, pH 7.4). The fractions containing purified sensor proteins were pooled and aliquots were snap-frozen at −80 °C. LC-MS analysis confirmed the correct and expected mass of the proteins with the overhanging amino acids Gly-Pro-Gly-Ser at the N-terminus derived from the PreScission protease site and the BamHI restriction site as described earlier [18].

### 2.2. Crystallization Conditions

For “crystallization condition A” (low Ca^2+^ concentration) crystallization was performed in a hanging drop, vapor diffusion set-up (Case16 structure A). Protein stock solution: 4.1 mg/mL Case16, 50 mM Tris-HCl, pH 7.4, 150 mM NaCl, 10 mM DTT. Reservoir solution: 50 mM imidazole, 1.9 M Na_2_malonate, pH 6.4. Drop: 3 μL protein solution and 1 μL reservoir solution. The reservoir volume was 0.5 mL. Incubation was performed at 20 °C. For “crystallization condition B” (high Ca^2+^ concentration) crystallization was performed in a hanging drop, vapor diffusion set-up (Case12 structure B). Protein stock solution: 7.6 mg/mL Case12, 50 mM Tris-HCl, pH 7.4, 150 mM NaCl. Seed stock solution: 200 mM CaCl_2_, 20% PEG-3350. Reservoir solution: 100 mM Tris-HCl, pH = 5.5, 100 mM (NH_4_)_2_SO_4_, 21% PEG-3350. Drop: 1 μL protein solution, 1 μL reservoir solution and 1 μL seed stock solution. The reservoir volume was 0.5 mL. Incubation was performed at 20 °C.

### 2.3. Data Collection and Analysis

The data set of Case16 crystals (“crystallization condition A”) was collected to a resolution of 2.35 Å at the PXII (X10SA) beam line at the Swiss Light Source (SLS) in Villigen, Switzerland. This beam line was equipped with a MARCCD detector and cryo-stream to keep the crystals at 100 K. Images were collected with 0.5° oscillation each and a crystal-to-detector distance of 200 mm. The dataset of the Case12 crystal (“crystallization condition B”) was collected to a resolution of 2.6 Å on a Rigaku FRE generator equipped with a MAR Image plate and an Oxford Cryo-system. Raw diffraction data were processed and scaled with XDS/Xscale [19] within the APRV program package [20]. The data collection statistics can be found in Table 1.

### 2.4. Structure Determination and Refinement

Case16 structure A (at low Ca^2+^ concentration) was solved using the molecular replacement program MOLREP [21] with GFP as a search model. The distorted CaM was traced by placing polyalanine α-helices into the residual electron density, followed by several cycles of refinement and gradually completing the model. For Case12 structure B (at high Ca^2+^ concentration) cpFP and CaM were localized independently by molecular replacement. Model building was performed using the COOT program [22]. The model was refined by the program REFMAC [23] which is part of the CCP4 suite (Collaborative Computational Project 1994). The refinement statistics is given in the Table 1. Figures were made using the program PYMOL (DeLano Scientific LLC, Palo Alto, CA, USA).

### 2.5. Characterization of the Oligomerization State of Ca^2+^ Sensors with Multi Angle Light Scattering (MALS)

Case12 and Case16 sensors were analyzed at concentrations of 4 mg/mL or 16 mg/mL. For higher concentrations the protein samples were concentrated using an Amicon Ultra 4 10kDa MWCO membrane (Millipore). Solutions containing 100 mM EGTA or 100 mM CaCl_2_ were added to a final concentration of 5 mM resulting in Ca^2+^-free or Ca^2+^-saturated states of the sensor, respectively. After further incubation for 1 h, 200 μL of the protein solution were loaded onto a Superdex 200 HR30/10 column mounted on an AEKTA Purifier 100 chromatography system. The running buffer contained 50 mM Tris-HCl, 300 mM NaCl, pH 7.4 and either 5 mM EGTA or 1 mM CaCl_2,_ depending on the sensor form to be analyzed. The size exclusion column was connected serially with a MiniDawn Tri-Star multi-angle light scattering detector (Wyatt Technologies) and RI-71 refractive index detector. This set-up allowed a direct on-line determination of the oligomerization state (*i.e.*, monomer *vs*. dimer) of the eluted protein.

### 2.6. Testing the Sensitivity of Case12 and Case16 Sensors to Free CaM or M13-peptide

Each of the three proteins Case 12, Case 16 and bovine CaM (Calbiochem) was incubated in TBS buffer together with 10 mM EGTA and subsequently dialyzed extensively against pure TBS to obtain the Ca^2+^-free form of the proteins excluding the Ca^2+^-chelating agent EGTA. M13-peptide (Ac-SSRRKWQKTGHAVRAIGRLSS-NH_2_; Biosyntan GmbH) was dissolved in PBS. The final concentrations for different components were 10 μM for both Ca^2+^ sensor proteins, 50 μM for CaM and 200 μM for M13-peptide. The solutions containing CaM or M13 peptide were diluted serially in 2-fold steps into a 384 low volume black multi-well plate (Greiner), 20 μL in each well. An equal volume of the samples, containing either Case 12 or Case 16 was then added. The final concentration of the sensors was fixed at 5 μM and ranged between 0.2–25 μM for CaM and 0.8–100 μM for M13-peptide, respectively. Ca^2+^ sensors diluted in pure TBS served as “zero value” and all samples were mixed in triplicates. The fluorescence intensity of the samples was measured using an Envision multilabel plate reader (Perkin Elmer) with the excitation wavelength set at 485 nm and the emission wavelength set at 535 nm. The first measurement cycle was performed with the proteins in absence of Ca^2+^, giving the “low value (L)”. The second measurement cycle was performed after addition of 4 μL 0.1 M CaCl_2_ (final concentration 10 mM) and incubation for 15 min at room temperature resulting in the “high value (H)”.

## 3. Results and Discussion

### 3.1. Structure of Case16 Sensor Freezed in Its “half Ca^2+^-bound” State

Earlier we described circularly permuted GFP (cpGFP)-based Ca^2+^ sensors Case12 and Case16 with superior dynamic ranges of up to 12-fold and 16.5-fold increase in green fluorescence between Ca^2+^-free and Ca^2+^-saturated forms [7]. The overall structure of these sensors is a monomer consisting of cpGFP “core” in its typical β-barrel shape inserted between an M13-peptide and CaM-domains.

**Figure 1.**
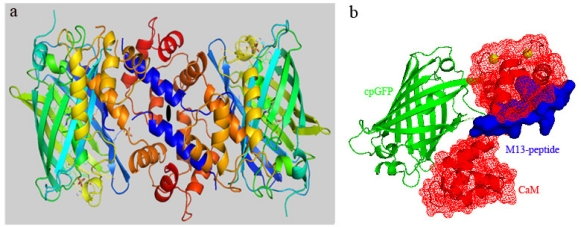
Schematic representation of Case16 structure A. (a) Case16 crystallized as a monomer in the asymmetric unit (in rainbow coloring mode: N-terminus blue, C-terminus red). The crystallographic contact between C-terminal lobes of CaM of the two Case16 molecules is a result of a packing interaction rather than a binding interaction. (b) Domain representation of Case16 structure A. M13-peptide is colored blue, cpGFP is green and N- and C-terminal lobes of CaM are shown red. Ca^2+^ ions are shown as yellow spheres.

In the present study Case16 sensor protein was crystallized at a low Ca^2+^ concentration (see Experimental Section, “*crystallization condition A”* and Table 1). No Ca^2+^ was added in the protein stock solution and in the reservoir solution. At the same time, no special Ca^2+^-free reagents or Ca^2+^ purification steps were used. Therefore the starting solution for crystallization contained background levels of Ca^2+^ coming from Na_2_malonate, NaCl and other reagents. It was reported earlier that adding an excess of a Ca^2+^ chelator (EGTA) did not yield a Ca^2+^-free structure and still produced crystals at dimeric Ca^2+^-bound form [15]. However, under “crystallization condition A” we were able to freeze the Case16 sensor in its “half Ca^2+^-bound state”. The crystal structure of Case16 (Case16 structure A, refined to 2.35 Å resolution) formed a monomer in the asymmetric unit (Figure 1a).

It was reported earlier that CaM possesses 4 EF-hand Ca^2+^-binding sites with differing Ca^2+^-binding affinities [24,25]. While the high-affinity C-terminal pair of sites is occupied at relatively low Ca^2+^ concentrations (below 500 nM), the N-terminal pair is occupied only at higher Ca^2+^ concentrations (above 500 nM). Therefore the initial Ca^2+^-dependent fluorescent response of the sensor in 100–500 nM range of Ca^2+^ can be determined by the structural rearrangements in response to filling of the first two high-affinity Ca^2+^-binding pockets of the C-terminal part of CaM. In agreement with this data, the structure A of Case16 reveals that only the two C-terminal Ca^2+^-binding sites of CaM are occupied with Ca^2+^ whereas both of its N-terminal sites are Ca^2+^-free. It is assumed that this structure of Case16 corresponds to the first stage of the fluorescent response of the sensor to low (below 500 nM) Ca^2+^ concentrations.

It is also important to note that Case16 structure A demonstrates an unexpected binding mode of CaM and M13-peptide. The CaM domain is in its open extended conformation and the M13-peptide was bound only to the C-terminal lobe of CaM (Figure 1b). Most likely, this particular CaM-M13 binding mode is mediated by the two complexed Ca^2+^ ions and an aromatic residue of the M13-peptide (Trp^9^) penetrating into a deep pocket of the C-terminal lobe of CaM. The N- and C-terminal CaM lobes of the previously reported structure [26] (PDB Access code = 1 CDL) align with an rmsd (root mean square deviation) of 4.60 Å (72 C-alpha atoms) and 1.00 Å (67 C-alpha atoms), respectively. This shows that the structure of the C-teminal lobe aligns well, while the N-terminal lobe of CaM shows a completely different arrangement, not only due to the open conformation, but also due to conformational changes induced by the missing Ca^2+^ ions.

In the wild type *Aequorea victoria* GFP (wtGFP) amino acid residues 143–152 form the 7th β-strand of the β-barrel. In Case16 the GFP moiety is circularly permuted leading to a break between positions 145 and 148. This break shortens the 7th β-strand in comparison to wtGFP and the new N- and C-termini of cpGFP are connected to M13- and CaM-domains of the sensor, respectively (a.a. numbering corresponds to wtGFP, see Supplementary Figure 1). Local rearrangements that are caused by the binding of the M13 peptide to CaM can influence the stability of this β-strand and thus may alter the fluorescent properties of the sensor molecule. Based on the resolved structure of Case16 we propose that binding of the M13-peptide to the C-terminal lobe of CaM in the context of the sensor stabilizes the arrangement of the 7th β-strand of GFP in its natural position. In other words, amino acid residues corresponding to positions 148–150 of the GFP should protrude out of the β-barrel as was predicted for the reconstructed Ca^2+^-free structure of GCaMP2 (PDB Access code = 3EKJ) [15]. The N-terminal lobe of CaM is in close contact with cpGFP and should further “clasp” and stabilize the 7th strand of GFP β-barrel (Figure 1). Therefore, the most likely explanation for the markedly increased fluorescence response of Case16 to low Ca^2+^ concentrations is that interactions between the CaM and the M13-peptide trigger chromophore conversion to the deprotonated high-fluorescent state. This hypothesis is in good agreement with conclusions from the Ca^2+^-saturated structure of GCaMP2 [15], which suggests that the key role of Ca^2+^-bound CaM is to reduce the solvent access to the chromophore. The structural changes that occur between the half Ca^2+^-saturated and Ca^2+^-saturated forms likely induce further changes in the chromophore environment that result in the fluorescent response to high Ca^2+^ concentrations. We believe that the obtained data can be extrapolated to all cpGFP-based FCIPs since they share high sequence homology (Supplementary Figure 1).

### 3.2. Chromophore Environment in Case16 Structure A

**Figure 2.**
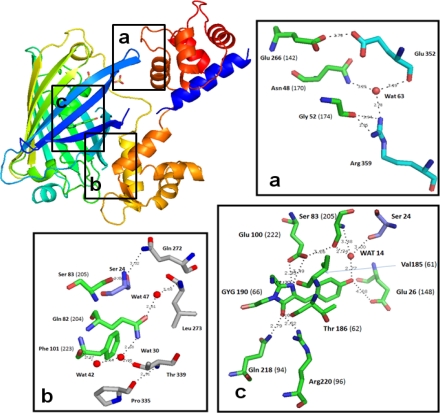
Ribbon diagram and key residues of the chromophore environment of Case16 structure A (colored in rainbow mode: N-terminus blue, C-terminus red). Insert **“a”** shows the loose contact of cpGFP with the C-terminal CaM lobe. Insert **“b”** shows the tight contact with the N-terminal lobe which is dominated by hydrophobic interactions. Insert **“c”** shows the local environment of cpGFP chromophore. Residue numbers indicated in brackets refer to wtGFP numbering. Residues with carbon atoms in green belong to cpGFP domain, residues with carbon atoms in cyan belong to CaM-domain and residues with carbon atoms in blue belong to M13-peptide.

Case16 structure A demonstrates that Glu^148^ contacts the cpGFP chromophore directly (Figure 2), which is consistent with our previous conclusions based on site-specific mutagenesis [7]. Therefore it can influence significantly the fluorescent properties of the sensor. In contrast, Ser^145^ is located rather distant from the chromophore (Figure 2, Supplementary Figure 2), refuting our previous hypothesis that both residues (Ser^145^ and Glu^148^) are in direct contact with the chromophore [7]. The present results demonstrate that Ser^145^ is instead a part of the peptide linker joining cpGFP with CaM-domain. To confirm this we performed site-directed mutagenesis to generate the mutant variant Case16-Ser145Ala. *In vitro* tests demonstrated that Case16-Ser145Ala was also characterized by high-contrast Ca^2+^-dependent fluorescent response, (up to 10-fold increase of green fluorescence upon Ca^2+^-saturation) which is close to the response of Case12 (data not shown). These results indicate that the amino acid residue 145 does not contact cpGFP chromophore within cpGFP-based Ca^2+^ sensor constructs directly. This is consistent with the previously reported Ca^2+^-saturated structures of Ca^2+^ sensor GCaMP2, demonstrating that Thr^145^ is not in close proximity to the chromophore [14,15].

Also of note is that Tyr^143^ is the last residue of the sensor construct which keeps its original location in the β-barrel as compared to GFP, while the NSRDQL stretch acts as a linker between cpGFP and CaM-domains. The side chain of Tyr^143^ protrudes out of the beta-barrel and thus can not interact with the chromophore of cpGFP in this structure.

### 3.3. At high Concentrations Ca^2+^ Sensors Can form Non-Functional Homodimers in Presence of Saturating Ca^2+^ Concentrations

**Figure 3.**
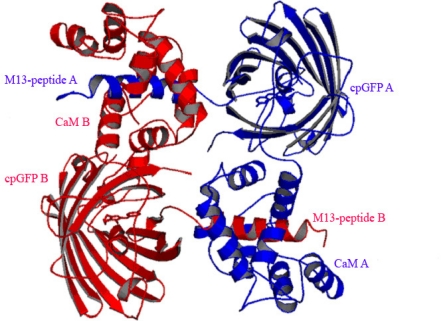
Ribbon diagram of Case12 structure B. Two Case12 (chains A and B are indicated in blue and red, respectively) that form intermolecular complexes between M13-peptide of chain A and CaM-domain of chain B (and *vice versa*) are shown.

Crystallization condition B (see Experimental Section) was used to generate a Ca^2+^-saturated crystal of another high-contrast Ca^2+^ sensor variant, Case12 [7]. The 3D-structure of Case12 at high Ca^2+^ concentration (Case12 structure B) was refined to a 2.6 Å resolution (Table 1) and revealed that the binding mode of the M13-peptide to the CaM-domain was similar to the recently reported Ca^2+^-saturated dimeric crystal structures of GCaMP2 [14,15]. Case12 structure B is a dimer in the asymmetric unit where the M13-peptide is bound to the CaM-moiety of the neighboring molecule (Figure 3). In contrast to the Case16 structure A, both lobes of CaM are bound to M13-peptide of a neighboring sensor molecule. As was expected for the Ca^2+^-saturated form of the sensor, all four Ca^2+^-binding pockets of CaM are occupied by Ca^2+^ ions. The binding mode between CaM and the M13- peptide is similar to that reported in [26] (1CDL)). The alignment of both structures has an rmsd of 0.91 for the whole CaM-domain (127 Cα positions).

**Figure 4.**
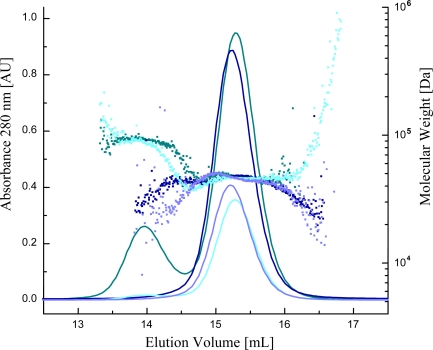
Gel-filtration light-scattering analysis of Case12. The solid lines represent the signals of the eluted proteins at 280 nm and the small squares represent the calculated molecular masses obtained from the multi-angle light scattering measurements. The colors are coded as following: dark green—Case12 at 16 mg/mL + Ca^2+^; dark blue—Case12 at 16 mg/mL + EGTA; cyan—Case12 at 4 mg/mL + Ca^2+^; light blue—Case12 at 4 mg/mL + EGTA.

To estimate the rate of homodimerization of Case12 and Case16 we performed *in vitro* measurements using static multi-angle light scattering in conjunction with gel filtration at moderate and high sensor protein concentrations in the presence/absence of Ca^2+^ (Figure 4 and Table 2). For the first set of experiments we used Case12 and Case16 at a concentration of 4 mg/mL. At this concentration, in the presence of 1 mM Ca^2+^ or EGTA, both sensors were characterized by a homogeneous molecular mass of about 45 kDa, corresponding to the monomeric form. Similar results were obtained for Case12 and Case16 at high concentration (16 mg/mL) in presence of EGTA. In contrast, at high concentration (16 mg/mL) in presence of 1 mM Ca^2+^ a second distinct molecular mass of about 90 kDa was observed indicating dimerization for both sensors. These light-scattering experiments demonstrate that in the presence of Ca^2+^ Case12 and Case16 can form dimers at high sensor protein concentrations (Figure 4 and Table 2).

Case12 and Case16 did not show any dimerization taken at 4 mg/mL or 16 mg/mL in the absence of Ca^2+^. However both indicators tend to dimerize at concentration of 16 mg/mL in presence of 1 mM Ca^2+^. At concentration of 4 mg/mL both sensors formed only low rate of the dimeric form in presence of 1 mM Ca^2+^.

Importantly the maximum fluorescent response of Case12 and Case16 *in vitro* does not require high concentrations of the sensor (to test the Ca^2+^ response *in vitro* we usually use sensor protein concentrations 0.01–0.1 mg/mL) while dimerization only becomes significant at much higher protein concentrations (16 mg/mL). Therefore the detected fluorescent response of the sensor within the whole range of the monitored Ca^2+^ concentrations is induced by its monomeric form rather than its homodimer. The obtained Case12 structure B most probably resembles the non-functional homodimer and should not be interpreted as an example of the active Ca^2+^-saturated state of the sensor. Similar data were reported earlier for GCaMP2 Ca^2+^-saturated dimeric form [14,15].

In general, high expression levels of FCIPs are desirable for monitoring of Ca^2+^ changes *in vivo*. It should be noted that initial attempts to generate functional transgenic mouse lines with constitutive expression of FCIPs were largely unsuccessful. The possible explanation is that at low expression levels typically achieved in transgenic mice (40–190 nM) a substantial fraction of the genetically encoded sensor becomes immobilized and unresponsive. Such low FCIP expression levels were insufficient to monitor single-cell activity and to perform single-spike detection in neurons [27]. High levels of expression are often more easily attainable using virus-mediated gene transfer because of the presence of multiple copies of the viral genome. Recombinant adeno-associated virus (rAVV) gene transfer was used to drive robust (tens of μM) expression of an improved fluorescence resonance energy transfer-based FCIP D3cpv in neurons that allowed single-spike detection [28]. The rAVV expression system permits gene delivery in a wide range of animals and should be useful for targeting most neuronal cell types in the brain. For example, using rAAV delivery strategy of Case12 it was demonstrated that astroglia is the main cellular substrate of angiotensin-(1-7) action in rat rostral ventro-lateral medulla, indicating that high expression level of FCIP is crucial for its efficient functioning [29].

On the other hand, our data indicate that homodimer formation is favored at high concentrations of GCaMP-like sensors. Although such homodimerization is negligible at moderate sensor concentration (4 mg/mL or approximately 85 μM, which corresponds to the level of FCIP expression in neurons by means of rAVV delivery), it becomes significant at 4-fold higher concentration, that can be also potentially achieved locally in living cells at high expression levels, especially for the FCIP versions targeted to the specific cellular compartments. This effect should be taken into account for the future design of improved Ca^2+^ sensors.

### 3.4. The Dependence of Case12 and Case16 Fluorescent Response on High Concentrations of Free CaM or M13-Peptide

Apart from homodimer formation, CaM/M13-based FCIPs can also interact with free CaM and CaM-binding proteins that may interfere with their functions. In order to decrease interactions with putative intracellular targets, Palmer and coworkers successfully reengineered the binding interface of CaM and M13-peptide by computational design and generated a mutant CaM-M13 pair that was unaffected by large concentrations of excess CaM and was used to obtain improved FCIP variants [30]. However, no titration was performed using excess M13-peptide, thus it remained unclear whether this design also reduced the sensitivity to numerous CaM-binding partners.

**Figure 5.**
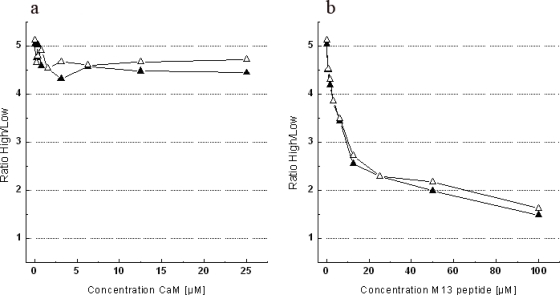
Titration experiments of Case12 and Case16 with free M13-peptide and CaM. (a) The titration of Case12 and Case16 with free CaM. (b) The titration of Case12 and Case16 with free M13-peptide. Filled symbols correspond to the values obtained for Case12 and open symbols for Case16.

We performed experiments where Case12 or Case16 were mixed with either free CaM or M13-peptide at various concentrations in order to check whether they may disrupt the intramolecular binding of M13-peptide to CaM within the sensor and thus decrease its dynamic range (for the experimental setup see Experimental Section). For the titration experiments, free M13-peptide and CaM were incubated with the sensor proteins at a final concentration of 5 μM (corresponding to 0.25 mg/mL). Purified Case12 and Case16 proteins were titrated *in vitro* with 5-fold molar excess of CaM or 20-fold molar excess of M13-peptide, respectively. Under our conditions CaM did not inhibit Ca^2+^-dependent fluorescent response of both sensor variants. In contrast, addition of M13-peptide significantly inhibited the sensor response even at low concentrations (Figure 5). Based on this data we hypothesize that interaction of the sensor construct with free M13-peptide is spatially and energetically more favorable than the intramolecular binding between M13-peptide and CaM. It remains unclear why the analogous effect was not observed with free CaM. A possible explanation is that the larger size and/or electrostatic repulsion of CaM in the sensor construct interferes with the binding of added CaM molecule to the M13-peptide of the sensor construct.

## 4. Conclusions

Using low Ca^2+^ concentration we were able to freeze the GCaMP-like Ca^2+^ sensor Case16 in its “half Ca^2+^-bound state” where only two C-terminal Ca^2+^-binding sites of CaM were occupied with Ca^2+^. Most likely this Case16 structure A corresponds to the first stage of the fluorescent response of the sensor at low Ca^2+^ concentrations. In contrast, the crystal structure of a closely related Ca^2+^ sensor Case12 (Case12 structure B) in presence of high Ca^2+^ concentrations shows that such sensors can dimerize. As shown in our light-scattering experiments, the formation of such a homodimer is favored at high sensor protein concentrations that could potentially be achieved locally in living cells with high sensor expression levels. We believe that the obtained data can be extrapolated to other cpGFP-based GCaMP-like Ca^2+^-sensors due to their high degree of homology (see Supplementary Figure 1 for a sequence alignment), and that our results will enable design of new genetically encoded FCIPs with improved characteristics.

## Supplementary Data

Supplementary Figure 1. Amino acid alignment of wild type *A. victoria* GFP, Case12, Case16, GCaMP2 and Calmodulin.
Structurally important regions are highlighted grey, beta-strands are shown with arrows and alpha-helixes with ribbons. Buried residues are shaded. Parts of the sensor constructs corresponding to Calmodulin are marked blue. Amino acid residues differing among the sensor constructs are shown yellow. Overall alignment numbering corresponds to that of avGFP.

Supplementary Figure 2. Diagram of Case16 structure A and its superposition with Case12 structure B.
(a) Ribbon diagram of Case16 structure B. Glu^148^ residue is in close proximity to cpGFP chromophore (green ribbon) whereas Ser^145^ residue is rather distant from the chromophore being part of the peptide linker joining cpGFP with CaM-domain (red ribbon). The N-terminal M13-peptide is shown as a blue ribbon.
(b) Superposition of Case16 structure A (yellow) and Case 12 structure B (red). In Case16 structure A Glu^148(26)^ in the 7th β-strand of cpGFP is pointing inside the β-barrel contacting chromophore directly. In contrast, the 7th β-strand of cpGFP in Case12 structure B is distorted and Glu^148(26)^ points to the exterior of the β-barrel opening it towards the solvent area.

## Figures and Tables

**Table 1.** Data collection and refinement statistics.

	**Case16 structure A**	**Case12 structure B**
**Data collection^a^**		
X-ray source	Swiss Light Source (PX2)	Rigaku FRE
Detector type	CCDCHESS-3072-SLS-PX2 225 mm	MAR345-2300-PSU-345 mm
Wavelength (Å)	0.99990	1.54179
Space group	P2_1_2_1_2	P2_1_
Cell dimensions		
a, b. c (Å)	92.5, 106.9, 43.3	46.4, 101.6, 82.2
α, β, γ (deg)	90.0, 90.0, 90.0	90.0, 91.5, 90.0
Resolution	92.50–2.35 (2.54–2.35)	100.00–2.60 (2.71–2.60)
Rsym (%)	8.8 (57.7)	10.1 (51.7)
I/sig(I) (%)	14.4 (3.3)	12.0 (2.8)
Completeness (%)	98.7 (90.1)	99.8 (99.8)
Completeness (highest shell I/sig(I) > 3)(%)	38.7	31.3
Redundancy (%)	6.9 (6.9)	3.7 (3.7)
Number of observed reflections	128,528	87,933
Number of unique Reflections	18,555	23,464
Wilson Bfactor (Å^2^)	49.6	41.9
**Refinement^b^**		
Resolution range (Å)	70.01–2.35 (2.411–2.35)	50.83–2.60 (2.667–2.60)
Completeness (%)	99.94 (100.0)	99.83 (99.77)
Number of reflections	17.624 (1,278)	22.288 (1,626)
R value	0.1995	0.245
R free	0.2730	0.316
R free test set size (%)	5.0	5.0
R value	0.217	0.340
R free	0.348	0.453
Number of atoms	3169	6434
Mean B (Å^2^)	51.129	22.432
PDB ID	3077	3078
Estimated coordinate error based on		
R value	0.380	0.939
R free	0.278	0.450
ML	0.212	0.376
B ML (Å^2^)	16.931	24.100
Bond lengths (Å)	0.022	0.015
Bond angles (deg)	1.978°	1.726°

a Highest resolution shell is shown in parenthesis.

b No sigma cutoffs.

**Table 2.** Dimerization rate of Case12 and Case16 in the presence/absence of Ca^2+^.

**Sensor protein concentration**	**Ca^2+^ concentration**	**Case12**	**Case16**

4 mg/mL	1 mM	3% dimer	2% dimer
4 mg/mL	0 mM (5 mM EGTA)	0% dimer	0% dimer
16 mg/mL	1 mM	22% dimer	26% dimer
16 mg/mL	0 mM (5 mM EGTA)	0% dimer	0% dimer

## References

[1] Miyawaki A, Llopis J, Heim R, McCaffery JM, Adams JA, Ikura M, Tsien RY (1997). Fluorescent indicators for Ca^2+^ based on green fluorescent proteins and calmodulin. Nature.

[2] Baird GS, Zacharias DA, Tsien RY (1999). Circular permutation and receptor insertion within green fluorescent proteins. Proc. Natl. Acad. Sci. USA.

[3] Nagai T, Yamada S, Tominaga T, Ichikawa M, Miyawaki A (2004). Expanded dynamic range of fluorescent indicators for Ca^2+^ by circularly permuted yellow fluorescent proteins. Proc. Natl. Acad. Sci. USA.

[4] Nakai J, Ohkura M, Imoto K (2001). A high signal-to-noise Ca^2+^ probe composed of a single green fluorescent protein. Nat. Biotechnol.

[5] Ohkura M, Matsuzaki M, Kasai H, Imoto K, Nakai J (2005). Genetically encoded bright Ca^2+^ probe applicable for dynamic Ca^2+^ imaging of dendritic spines. Analyt. Chem.

[6] Tallini YN, Ohkura M, Choi BR, Ji G, Imoto K, Doran R, Lee J, Plan P, Wilson J, Xin HB, Sanbe A, Gulick J, Mathai J, Robbins J, Salama G, Nakai J, Kotlikoff MI (2006). Imaging cellular signals in the heart *in vivo*: Cardiac expression of the high-signal Ca^2+^ indicator GCaMP2. Proc. Natl. Acad. Sci. USA.

[7] Souslova EA, Belousov VV, Lock JG, Strömblad S, Kasparov S, Bolshakov AP, Pinelis VG, Labas YA, Lukyanov S, Mayr LM, Chudakov DM (2007). Single fluorescent protein-based Ca^2+^ sensors with increased dynamic range. BMC Biotechnol.

[8] Tian L, Hires SA, Mao T, Huber D, Chiappe ME, Chalasani SH, Petreanu L, Akerboom J, McKinney SA, Schreiter ER, Bargmann CI, Jayaraman V, Svoboda K, Looger LL (2009). Imaging neural activity in worms, flies and mice with improved GCaMP calcium indicators. Nat. Methods.

[9] Heim N, Griesbeck O (2004). Genetically encoded indicators of cellular calcium dynamics based on troponin C and green fluorescent protein. J. Biol. Chem.

[10] Mank M, Reiff DF, Heim N, Friedrich MW, Borst A, Griesbeck O (2006). A FRET-based calcium biosensor with fast signal kinetics and high fluorescence change. Biophys. J.

[11] Mank M, Santos AF, Direnberger S, Mrsic-Flogel TD, Hofer SB, Stein V, Hendel T, Reiff DF, Levelt C, Borst A, Bonhoeffer T, Hübener M, Griesbeck O (2008). A genetically encoded calcium indicator for chronic *in vivo* two-photon imaging. Nat. Methods.

[12] Patterson GH, Lippincott-Schwartz J (2002). A photoactivatable GFP for selective photolabeling of proteins and cells. Science.

[13] Chudakov DM, Verkhusha VV, Staroverov DB, Souslova EA, Lukyanov S, Lukyanov KA (2004). Photoswitchable cyan fluorescent protein for protein tracking. Nat. Biotechnol.

[14] Wang Q, Shui B, Kotlikoff MI, Sondermann H (2008). Structural basis for calcium sensing by GCaMP2. Structure.

[15] Akerboom J, Rivera JD, Guilbe MM, Malavé EC, Hernandez HH, Tian L, Hires SA, Marvin JS, Looger LL, Schreiter ER (2009). Crystal structures of the GCaMP calcium sensor reveal the mechanism of fluorescence signal change and aid rational design. J. Biol. Chem.

[16] Palmer AE, Giacomello M, Kortemme T, Hires SA, Lev-Ram V, Baker D, Tsien RY (2006). Ca^2+^ indicators based on computationally redesigned calmodulin-peptide pairs. Chem. Biol.

[17] Zeng G (1998). Sticky-end PCR: new method for subcloning. Biotechniques.

[18] Freuler F, Stettler T, Meyerhofer M, Leder L, Mayr LM (2008). Development of a novel Gateway-based vector system for efficient, multiparallel protein expression in Escherichia coli. Protein Expr. Purif.

[19] Kabsch W (1988). Automatic indexing of rotation diffraction patterns. J. Appl. Crystallogr.

[20] Kroemer M, Dreyer MK, Wendt KU (2004). APRV—A program for automated data processing, refinement and visualization. Acta Crystallogr. D. Biol. Crystallogr.

[21] Vagin AA, Teplyakov A (1997). MOLREP: An automated program for molecular replacement. J. Appl. Crystallogr.

[22] Emsley P, Cowtan K (2004). COOT: model-building tools for molecular graphics. Acta Crystallogr. D. Biol. Crystallogr.

[23] Murshudov GN, Vagin AA, Dodson EJ (1997). Refinement of macromolecular structures by the maximum-likelihood method. Acta Crystallogr. D. Biol. Crystallogr.

[24] Maune JF, Klee CB, Beckingham K (1992). Ca^2+^ binding and conformational change in two series of point mutations to the individual Ca(^2+^)-binding sites of calmodulin. J. Biol. Chem.

[25] Linse S, Helmersson A, Forsen S (1991). Calcium binding to calmodulin and its globular domains. J. Biol. Chem.

[26] Meador WE, Means AR, Quiocho FA (1992). Target enzyme recognition by calmodulin: 2.4 Å structure of a calmodulin-complex. Science.

[27] Hasan MT, Rainer WF, Euler T, Larkum ME, Giese G, Both M, Duebel J, Waters J, Bujard H, Griesbeck O, Tsien RY, Nagai T, Miyawaki A, Denk W (2004). Functional fluorescent Ca^2+^ indicator proteins in transgenic mice under TET control. PLoS Biol.

[28] Wallace DJ, Alten-Borghof SM, Astori S, Yang Y, Bausen M, Kugler S, Palmer AE, Tsien RY, Sprengel R, Kerr JND, Denk W, Hasan MT (2008). Single-spike detection *in vitro* and *in vivo* with a genetic Ca^2+^ sensor. Nat. Methods.

[29] Guo F, Liu B, Lane S, Souslova EA, Chudakov DM, Paton JFR, Kasparov S (2010). Astroglia is a possible cellular substrate of angiotensin-II effects in the rostral ventro-lateral medulla. Cardiovasc Res.

[30] Palmer AE, Giacomello M, Kortemme T, Hires SA, Lev-Ram V, Baker D, Tsien RY (2006). Ca^2+^ indicators based on computationally redesigned calmodulin-peptide pairs. Chem. Biol.

