# Supplementary figures and images for: The Structure of Ca2+ Sensor Case16 Reveals the Mechanism of Reaction to Low Ca2+ Concentrations
Source: Sensors (Basel). 2010 Aug 30;10(9):8143–60. doi: 10.3390/s100908143 (PMC3231237; doi:10.3390/s100908143)

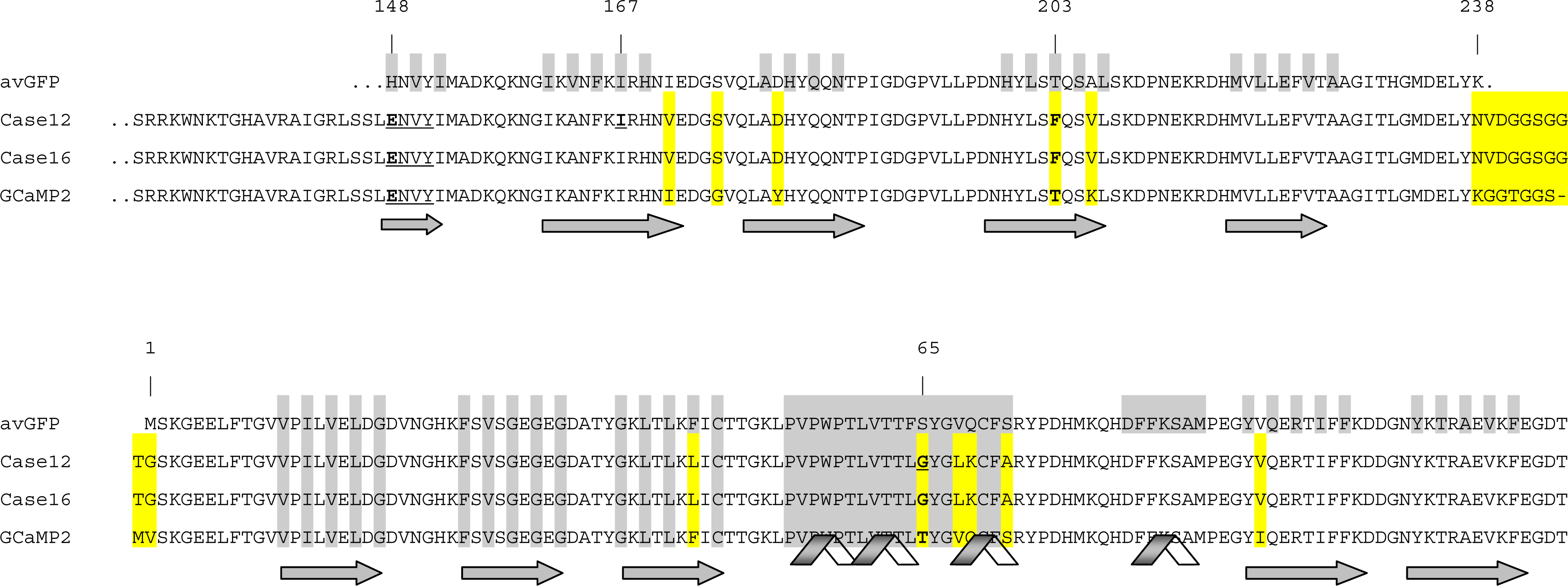

Supplement: Supplementary Figure 1. — Amino acid alignment of wild type A. victoria GFP, Case12, Case16, GCaMP2 and Calmodulin. Structurally important regions are highlighted grey, beta-strands are shown with arrows and alpha-helixes with ribbons. Buried residues are shaded. Parts of the sensor constructs corresponding to Calmodulin are marked blue. Amino acid residues differing among the sensor constructs are shown yellow. Overall alignment numbering corresponds to that of avGFP. [file sensors-10-08143-s001a.tif]

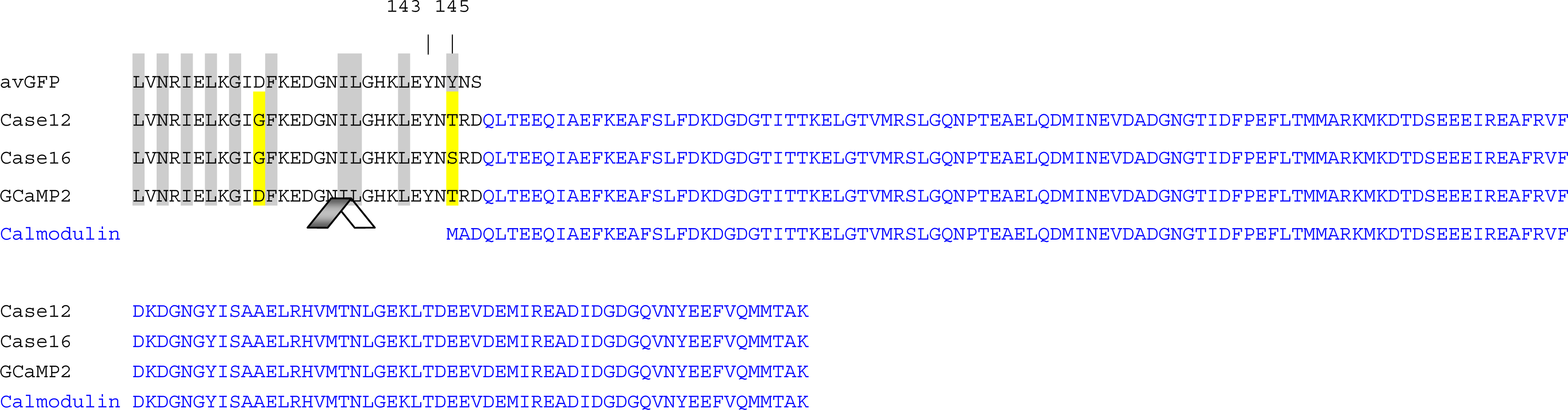

Supplement: Supplementary Figure 1. — Amino acid alignment of wild type A. victoria GFP, Case12, Case16, GCaMP2 and Calmodulin. Structurally important regions are highlighted grey, beta-strands are shown with arrows and alpha-helixes with ribbons. Buried residues are shaded. Parts of the sensor constructs corresponding to Calmodulin are marked blue. Amino acid residues differing among the sensor constructs are shown yellow. Overall alignment numbering corresponds to that of avGFP. [file sensors-10-08143-s001b.tif]

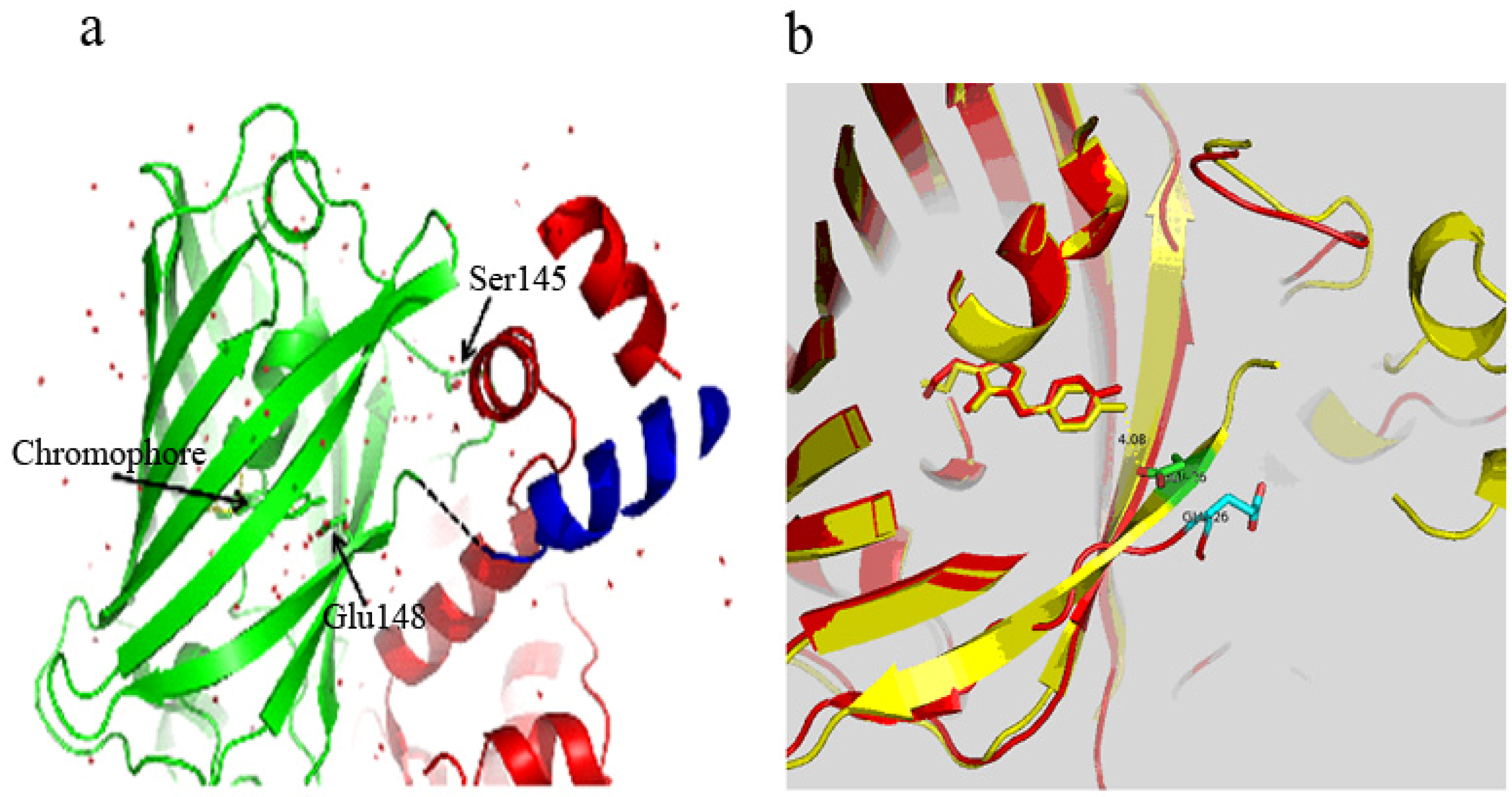

Supplement: Supplementary Figure 2. — Diagram of Case16 structure A and its superposition with Case12 structure B. (a) Ribbon diagram of Case16 structure B. Glu148 residue is in close proximity to cpGFP chromophore (green ribbon) whereas Ser145 residue is rather distant from the chromophore being part of the peptide linker joining cpGFP with CaM-domain (red ribbon). The N-terminal M13-peptide is shown as a blue ribbon. (b) Superposition of Case16 structure A (yellow) and Case 12 structure B (red). In Case16 structure A Glu148(26) in the 7th β-strand of cpGFP is pointing inside the β-barrel contacting chromophore directly. In contrast, the 7th β-strand of cpGFP in Case12 structure B is distorted and Glu148(26) points to the exterior of the β-barrel opening it towards the solvent area. [file sensors-10-08143-s002.tif]
